# Corneal Collagen Cross-Linking with Hypoosmolar Riboflavin Solution in Keratoconic Corneas

**DOI:** 10.1155/2014/754182

**Published:** 2014-08-14

**Authors:** Shaofeng Gu, Zhaoshan Fan, Lihua Wang, Xiangchen Tao, Yong Zhang, Guoying Mu

**Affiliations:** Department of Ophthalmology, Shandong Provincial Hospital, Shandong University, No. 324, Jing 5 Road, Huaiyin District, Jinan, Shandong 250021, China

## Abstract

*Purpose*. To report the 12-month outcomes of corneal collagen cross-linking (CXL) with a hypoosmolar riboflavin and ultraviolet-A (UVA) irradiation in thin corneas. *Methods*. Eight eyes underwent CXL using a hypoosmolar riboflavin solution after epithelial removal. The corrected distance visual acuity (CDVA), manifest refraction, the mean thinnest corneal thickness (MTCT), and the endothelial cell density (ECD) were evaluated before and 6 and 12 months after CXL. *Results*. The MTCT was 413.9 ± 12.4 *μ*m before treatment and reduced to 381.1 ± 7.3 *μ*m after the removal of the epithelium. After CXL, the thickness decreased to 410.3 ± 14.5 *μ*m at the last follow-up. Before treatment, the mean *K*-value of the apex of the keratoconus corneas was 58.7 ± 3.5 diopters and slightly decreased (57.7 ± 4.9 diopters) at 12 months. The mean CDVA was 0.54 ± 0.23 logarithm of the minimal angle of resolution before treatment and increased to 0.51 ± 0.21 logarithm at the last follow-up. The ECD was 2731.4 ± 191.8 cells/mm^2^ before treatment and was 2733.4 ± 222.6 cells/mm^2^ at 12 months after treatment. *Conclusions*. CXL with a hypoosmolar riboflavin in thin corneas seems to be a promising method for keratoconic eyes with the mean thinnest corneal thickness less than 400 *μ*m without epithelium.

## 1. Introduction

Keratoconus is a common disease of the cornea; the incidence in the general population is about 1/2000 [1]. It is characterized by progressive thinning and ectasia of the cornea. Corneal transplantation is inevitable leading to severe visual deterioration and corneal scarring in 20% of patients [2]. CXL is considered a promising and less invasive technique. Most studies suggest that CXL treatment improves the corneal rigidity [3, 4] and increases the corneal resistance to enzymatic digestion [5]. With UVA irradiation (365 nm) and riboflavin (as photosensitizer), new covalent bonds are induced between collagen molecules, fibers, and microfibrils by photosensitized oxidation [6]. The collagen fibrils diameter was increased and the proteoglycan area was reduced in the human keratoconus cornea after CXL treatment [7].

In 2003, Wollensak et al. pioneered CXL treatment to stop progression of keratoconus [8]. After that, a number of studies have demonstrated efficacy of arresting the progression of keratoconus by using CXL “standard” protocol [9–11]. According to “standard” protocol (epithelium removal, using isoosmolar 0.1% riboflavin solution, and UVA irradiation for 30 minutes), a minimal stromal thickness (without the corneal epithelium) of at least 400 *μ*m was required for safety [12, 13]. Unfortunately, in many cases of advanced progressive keratectasia, patients are often excluded from the CXL treatment because their corneal thickness is less than 400 *μ*m. To solve this problem, various modifications of the “standard” protocol have been attempted. Some studies found stabilization of the corneal ectasia by leaving the epithelium over the thinnest area intact, but the effect was mild [14, 15]. Hafezi and associates proposed an alternative treatment protocol by using hypoosmolar riboflavin solution (0.1% riboflavin in 0.9% saline instead of dextran) to swell the corneal stroma [16]. The results showed stabilization of keratometry and no complications by using hypoosmolar riboflavin solution [16, 17]. However, little has been known about the safety and efficacy of this treatment, and a failure case was reported by using hypoosmolar riboflavin solution in an extremely thin cornea [18].

In this study, we investigated the effectiveness and safety of CXL using hypoosmolar riboflavin solution and UVA for the treatment of keratoconus with the thinnest corneal thickness less than 400 *μ*m without epithelium.

## 2. Material and Methods

The study was approved by the ethics committee of the Shandong Provincial Hospital affiliated to Shandong University under the principles of the Helsinki Declaration. Informed consent was obtained from all study participants before the initiation of CXL treatment.

Patients with keratoconus were prospectively recruited from the Cornea Outpatient Clinic of Shandong Provincial Hospital affiliated to Shandong University. The inclusion criteria were progressive keratoconus (stages 1 to 3 keratoconus, according to the Krumeich classification [19]) documented within the past 12 months as evidenced by astigmatic refraction and/or topography, no previous ocular surgery, thinnest corneal thickness of less than 400 *μ*m (without epithelium), and no wearing of contact lenses before initial evaluation and treatment. Progression was considered if at least one or more of the following criteria were met: an increase of at least 1.00 diopter (D) in the steepest simulated keratometry reading (*K*max) derived from computerized videokeratography; an increase in astigmatism as determined by manifest subjective refraction of at least 1.00 D; an increase of 0.50 D in manifest refraction spherical equivalent. Exclusion criteria were a minimum corneal thickness >400 *μ*m, previous refractive surgery or other corneal surgery, a history of severe infections, or other corneal or ocular surface disease, and pregnancy or lactation (female patients).

Hypoosmolar riboflavin solution (0.1%) was generated by diluting vitamin B2-riboflavin-5-phosphate 0.5% (Shandong Fangming Pharmaceutical Limited by Share Ltd., Shandong, China) with physiological salt solution (sodium chloride 0.9% solution; 310 mOsmol/L; Sichuan Kelun Pharmaceutical Limited by Share Ltd., Sichuan, China). The procedure was performed under sterile conditions. After topical anesthesia using proparacaine hydrochloride 0.5% (Alcaine; Alcon Laboratories, Inc., Fort Worth, Texas, USA) eye drops, a lid speculum was inserted. The central cornea was contacted with a filter paper soaked with 20% alcohol for 60 seconds (the diameter was 9 mm), then the central 9 mm of cornea epithelium was removed with a blunt spatula (Asico AE2766). Deepithelialization was followed by measuring the corneal thickness with OCT (Cirrus HD-OCT 4000; Carl Zeiss Meditec Inc., Hacienda Drive, Dublin, USA) to validate that the thinnest thickness was less than 400 um. Hypoosmolar riboflavin solution (0.1%) was applied to the cornea every 3 minutes for 30 minutes. The corneal thickness was checked by OCT and hypoosmolar riboflavin solution was again administered until corneal thickness was more than 400 um at the thinnest point. A digital slit-lamp photograph (True Digital Slit Lamp SL DC-3; Topcon Corporation, Hasunuma-cho, Itabashi-Ku, Tokyo, Japan) was performed to ensure the appearance of riboflavin in the anterior chamber.

Then the eye was irradiated with UVA of 370 nm wavelength (UV-X illumination system version 1000, UVXTM, IROC AG, Zurich, Switzerland) at a working distance of 5 cm. An area with 9 mm diameter in the center of the cornea was irradiated with an irradiance of 3.0 mW/cm^2^. During the 30 minutes of irradiation, hypoosmolar riboflavin solution was applied every 3 minutes to maintain the riboflavin saturation in cornea stroma. At the end of the procedure, a combination of dexamethasone 0.1% and tobramycin 0.3% (Tobradex, Alcon Co. Ltd., USA) was administered 4 times daily in all patients and a bandage soft contact lens was applied until healing of the corneal epithelium was completed.

The MTCT was examined before and after removal of epithelium, after swelling, and 6 and 12 months after CXL by OCT device. The CDVA with glasses or contact lenses, manifest refraction (diopters; D), and corneal topography (Orbscan II; Bausch & Lomb Incorporated, Rochester, New York, United States) were assessed before and 6 and 12 months after the procedure. The ECD was acquired using a Specular Microscope (Specular Microscope SP-3000P; Topcon Corporation, Hasunuma-cho, Itabashi-Ku, Tokyo, Japan) before and 6 and 12 months after CXL.

Statistical evaluation of values before and 6 and 12 months after CXL was performed using the nonparametric test (Wilcoxon test) with SPSS software version 17 (SPSS GmbH Software, Munich, Germany). A *P* value below 0.05 was considered statistically significant.

## 3. Results

The analysis included 8 eyes of 8 patients (5 males and 3 females) with a mean age of 27.4 ± 3.6 years. All eyes had transparent corneas before the procedure.

**Figure 1:**
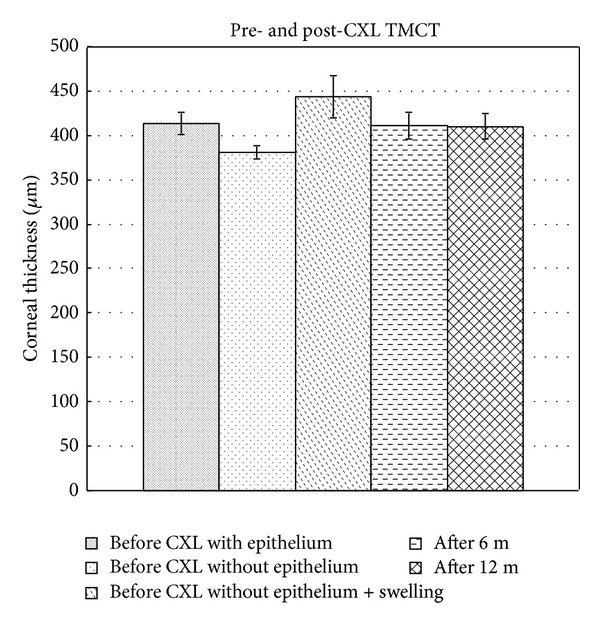
Bar graph showing the mean thinnest corneal thickness (MTCT) of patients with/without epithelium, after swelling by the hypoosmolar riboflavin solution and 6 months and 12 months after CXL.

Before treatment, the MTCT was 413.9 ± 12.4 *μ*m and 381.1 ± 7.3 *μ*m with and without epithelium. After swelling by the hypoosmolar riboflavin solution, the cornea thickness increased to 443.8 ± 23.9 *μ*m. The MTCT decreased at 6 months (411.5 ± 15.2 *μ*m) and remained stable at 12 months (410.3 ± 14.5 *μ*m) after treatment (Figure 1). Before and after operation the MTCT differences were not significant at 6 months (*P* = 0.4) and 12 months (*P* = 0.233).

**Figure 2:**
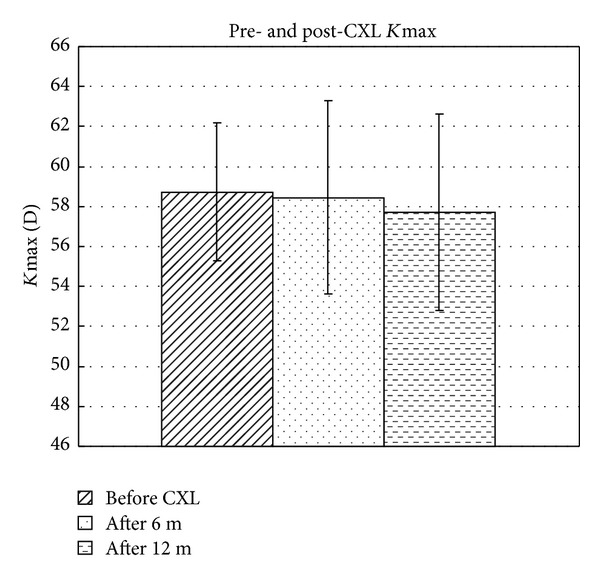
Bar graph showing the mean *K*-value at the apex of keratoconus (*K*max) before CXL and 6 months and 12 months after CXL with hypoosmolar riboflavin solution.

The mean *K*-value from the apex of the keratoconus was 58.7 ± 3.5 diopters before treatment. Six months after treatment, this value was maintained at 58.5 ± 4.8 (*P* = 0.674) and reduced to 57.7 ± 4.9 at 12 months (*P* = 0.611) (Figure 2). The differences between pre- and postoperative mean *K*max values were all not significant (all with *P* > 0.05).

**Figure 3:**
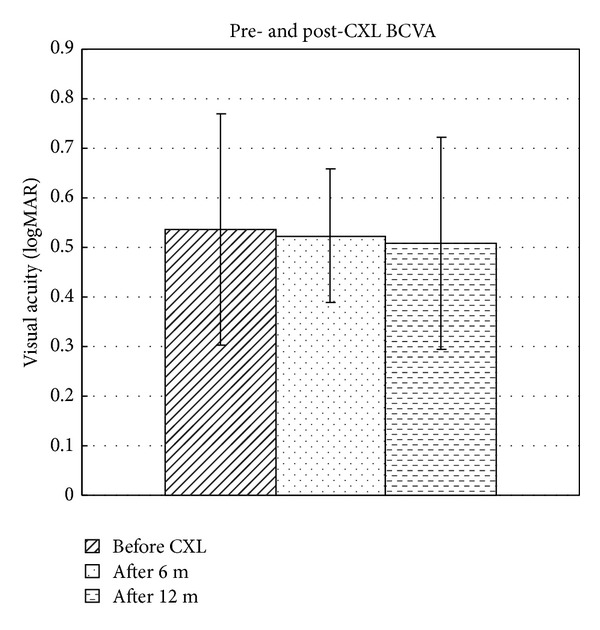
Bar graph showing the mean best corrected visual acuity (BCVA) of patients before CXL and 6 months and 12 months after CXL with hypoosmolar riboflavin solution. logMAR: logarithm of the minimal angle of resolution.

The mean CDVA at the time of the treatment was 0.54 ± 0.23 logarithm of the minimal angle of resolution and increased to 0.52 ± 0.13  (*P* = 1) at 6 months and 0.51 ± 0.21 (*P* = 0.285) at 12 months after treatment (Figure 3). The mean CDVA showed no significant change at these follow-up visits compared to pre-CXL values (all with *P* > 0.05). At the 12-month follow-up, 25% (2 of 8 eyes) gained at least 1 Snellen line and 62.5% (5 of 8 eyes) showed a stable CDVA.

**Figure 4:**
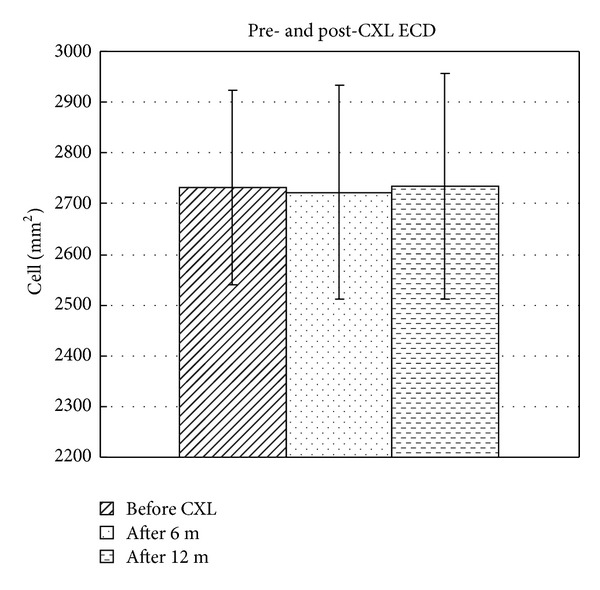
Bar graph showing the mean endothelial cell density (ECD) of patients before CXL and 6 months and 12 months after CXL with hypoosmolar riboflavin solution.

The mean ECD was 2731.4 ± 191.8 cells/mm^2^ before treatment and decreased to 2722.5 ± 211.5 cells/mm^2^ (*P* = 0.208) at 6 months after treatment and returned to 2733.4 ± 222.6 cells/mm^2^ (*P* = 0.327) at 12 months (Figure 4). There was no significant change in the mean ECD counts between values before and 6- and 12-month values after treatment (all with *P* > 0.05).

All corneal epithelial defects were closed 1 week after treatment. No corneal stroma infections were observed after treatment.

## 4. Discussion

CXL is a minimally invasive surgical technique, which stabilizes the progression of corneal ectasia and postpones the need of lamellar or penetrating keratoplasty [20–23]. Studies showed that CXL increase the diameter of the collagen fibers with most changes occurring in the anterior 300 *μ*m in the anterior stroma [22, 23]. As collagen bonds are established at a depth of 300 *μ*m in the anterior stroma, a minimum of 400 *μ*m stromal thickness is suggested for the safety of the endothelium [12, 13]. According to the criteria, patients with corneal stromal thickness less than 400 *μ*m would be excluded from treatment. In order to overcome this limitation, hypoosmolar riboflavin was used to increase corneal stromal thickness in CXL treatment for the safety [16].

In our study, we used this modified technique in 8 patients with thin corneas. Before treatment, the MTCT was 413.9 ± 12.4 *μ*m with epithelium and seemingly did not clearly fall under the thin category. After removal of epithelium, however, the MTCT reduced to 381.1 ± 7.3 *μ*m and fulfilled the inclusion criteria of our study. Results showed an improvement in the mean CDVA and a decrease in keratometry readings (the mean *K*max values) during the first year after treatment. The results of these parameters were similar to the previous studies [24–27]. We considered that these effects may be related to the corneal remodeling process after CXL. Studies found that CXL changed the abnormal keratoconic collagen fibrils distribution into normal fibrils distribution [7]. After CXL, the keratoconus corneal structure showed a modification in the collagen fibrils diameter, interfibrillar spacing, and the proteoglycan area. These modifications of the cornea stroma might result in a stable or decreased maximal keratometry readings and an improvement of CDVA. However, the *K*-value reduction achieved was rather small (−1.0 D) and not statistically significant. At our last follow-up, 25% (2 of 8 eyes) gained at least 1 Snellen line and 62.5% (5 of 8 eyes) showed a stable CDVA.

The mean ECD in our study remained stable at 6- and 12-month follow-up points. No adverse endothelial reaction and endothelial cell-related complications such as corneal edema were observed. These results were not consistent with the published literature following the CXL standard protocol in thin corneas, which resulted in a significant endothelial cell count loss postoperatively [28].

We observed that the MTCT was increased after swelling but decreased during the follow-up examination. A number of studies reported changes in corneal thickness after CXL treatment. Some studies showed that corneal thickness gradually increased after treatment and this increasing value did not reach preoperative reading at last follow-up [29–33]. Kanellopoulos and Asimellis reported the corneal thickness rebounding at three months [34]. In our study, however, we observed a decrease of corneal thickness after CXL, in agreement with a recent publication [35]. We thought the corneal deturgescence following treatment may be the reason for this decrease. It is well known that CXL could influence the swelling behavior of tissue [36, 37]. Wollensak et al. showed that the swelling behavior was dependent on the degree of CXL: the higher the CXL, the lower the corneal swelling behavior [38]. Alternatively, the reduced corneal thickness may be explained by the increase in endothelial pump activity or density induced by the treatment [33]. The reason and mechanism for these different results remained unexplained. Despite the decrease in the corneal thickness, we did not find a difference at each follow-up examination after CXL. We suggested that this decrease in corneal thickness did not imply a negative effect of CXL.

A study has shown that the epithelium was significantly thinner over areas of the corneal protrusion. Kanellopoulos et al. showed that the epithelium over an ectatic area was approximately 35 *μ*m in one example [39]. In our study, we found that the MTCT reduced to 381.1 ± 7.3 *μ*m after removal of epithelium and the decreased value (the thickness of the epithelium) was approximately 33 *μ*m, which was similar to Kanellopoulos' study. Kanellopoulos et al. also showed that the patients treated with CXL had epithelium thickness distributions that were similar to the normal group. They put forward a novel theory of “reactive” epithelial hyperplasia in biomechanically unstable corneas [39, 40].

Some limitations of our study included the limitation of the measurement method and a relatively small number of patients. Ultrasound pachymetry (UP) has been considered the gold standard for measurement of corneal thickness. In contrast to ultrasound pachymetry, OCT is a noninvasive, noncontact method. Publication suggested that corneal thickness measured with OCT may not be reliable because of the increased light scattering and absorption in swollen corneas [41]. However, studies have shown a high correlation between measurements of corneal thickness using both instruments [42, 43]. Kanellopoulos and Asimellis have investigated the application of anterior-segment OCT in various states of corneal transparency and found that at least compared to the Scheimpflug-principle devices (i.e., Pentacam) the OCT may be superior to Pentacam. The use of UP may be challenging due to the coupling required, although it is mostly “popular” in many countries [44]. In swollen cornea, the OCT has underestimated pachymetric measurements in some cases and overestimated ones in the others compared with the UP [45]. The reasons for these differences were not clear. On the other hand, our results may also be influenced by the small sample size. The number of eyes included in our study is small and this small sample size has less power to reach a stronger conclusion. More cases and long-term studies should address this finding in the future. Besides, further studies of corneal thickness changes by modalities, such as confocal microscopy and ultrasound pachymetry, may be warranted.

## 5. Conclusions

In essence, our results showed that CXL with a hypoosmolar riboflavin solution seemed to be a promising method for thinner corneas. A longer follow-up and larger patient series would be needed to evaluate the long-term effect and safety of the method in thin corneas.
